# Effects of tapering tumor necrosis factor inhibitor on the achievement of inactive disease in patients with axial spondyloarthritis: a nationwide cohort study

**DOI:** 10.1186/s13075-019-1943-6

**Published:** 2019-07-04

**Authors:** Jun Won Park, Hyoun-Ah Kim, Kichul Shin, Yong-Beom Park, Tae-Hwan Kim, Yeong Wook Song, Eun Young Lee

**Affiliations:** 1 Division of Rheumatology, Department of Internal Medicine, Seoul National University College of Medicine, 101 Daehak-ro, Jongno-gu, Seoul, Republic of Korea; 2 Department of Rheumatology, Ajou University School of Medicine, Suwon, Republic of Korea; 3 Division of Rheumatology, Department of Internal Medicine, SMG-SNU Boramae Medical Center, Seoul, Republic of Korea; 4 Division of Rheumatology, Department of Internal Medicine, Yonsei University College of Medicine, Seoul, Republic of Korea; 5 Department of Rheumatology, Hanyang University Hospital for Rheumatic Diseases, Seoul, Republic of Korea

**Keywords:** Spondyloarthritis, Tumor necrosis factor, Dose tapering, Inactive disease

## Abstract

**Objectives:**

To investigate the association between the extent of tapering tumor necrosis factor inhibitor (TNFi) and the likelihood of achieving inactive disease in patients with axial spondyloarthritis (axSpA)

**Methods:**

We analyzed 1575 1-year follow-up interval data of 776 axSpA patients treated with TNFi for more than 1 year in a nationwide observational cohort. The decision on tapering TNFi was made by patients and their physicians. We quantified TNFi used during interval as a dose quotient (DQ). The intervals were classified into the heavy-tapering (DQ < 50), mild-tapering (DQ 50–99), and control groups (DQ = 100). Outcome variables included achieving Ankylosing Spondylitis Disease Activity Score-inactive disease (ASDAS-ID) and major clinical response of Bath Ankylosing Spondylitis Disease Activity Index (BASDAI50) in the follow-up visit. The effects of TNFi tapering on the outcome were analyzed using the generalized estimating equation.

**Results:**

At the baseline visit, 91.1% of the patients showed a high disease activity (ASDAS-CRP ≥ 2.1). DQ of each interval was significantly influenced by the ASDAS-CRP measure in the prior follow-up (*P* < 0.001). ASDAS-ID was observed in 42.3% of the intervals. A multivariable analysis showed that the likelihood of outcome achievement was comparable between the control and mild-tapering groups, but significantly decreased in the heavy-tapering group (vs. the control group, adjusted OR = 0.28, [95% CI, 0.08–0.94]). In contrast, the likelihood to achieve BASDAI50 response was not different among the groups. In the subgroup of patients who reached ASDAS-ID 1 year after TNFi treatment (*n* = 327), ASDAS-ID was observed in 66.1% of the subsequent intervals, and only the mild-tapering group showed a likelihood of target maintenance comparable with that of the control group (adjusted OR = 1.25 [0.41–3.80]). This likelihood decreased with an increase in ASDAS-CRP.

**Conclusion:**

Mild tapering of TNFi has efficacy comparable with that of the standard-dose treatment for ASDAS-ID achievement in patients with axSpA.

**Electronic supplementary material:**

The online version of this article (10.1186/s13075-019-1943-6) contains supplementary material, which is available to authorized users.

## Background

The introduction of tumor necrosis factor inhibitor (TNFi) in the treatment of axial spondyloarthritis (axSpA) has considerably changed the outcome and prognosis of the disease. The efficacy of long-term TNFi treatment has been demonstrated by several randomized controlled trials and large cohort studies [1–7]. However, the long-term use of TNFi could increase economic burden on patients and the risk of infection and possibly some kinds of malignancy [7–9]. However, several patients continue TNFi treatment despite maintaining persistent stable disease activity because treatment discontinuation usually leads to flares [10].

To overcome this problem, some studies have focused on tapering TNFi in patients who maintain prolonged low disease activity and showed that this strategy could have comparable efficacy to that of the standard-dose TNFi treatment at a lower cost [11–14]. Based on the results of these studies, the European League Against Rheumatism (EULAR) in its recent guideline recommended that tapering TNFi can be considered for patients who achieve sustained remission [15]. Furthermore, a recommendation by the International Task Force emphasizes the “treat-to-target” strategy, according to which achieving clinical remission or inactive disease is the optimal target for the best outcome [16]. However, it is not clear whether tapering TNFi could also help to maintain the target. Moreover, information regarding patients in whom tapering should be tried and how it should be performed is limited, which is a hurdle for the application of the tapering strategy in real-world clinical settings.

Therefore, in this study, we aimed to (1) investigate whether tapering of TNFi and its extent could influence the likelihood of achieving inactive disease activity in patients with axSpA in a nationwide prospective cohort and (2) clarify the indication for tapering TNFi in real-world clinical settings by assessing the clinical factors that affect the probability of achieving inactive disease.

## Methods

### Study population

Data of the patients included in this study were collected from the Korean College of Rheumatology Biologics Registry (KOBIO) cohort, a nationwide cohort of patients with inflammatory arthritis receiving biologic disease-modifying antirheumatic drugs (bDMARDs) in daily clinical practice since January 2013 (NCT01965132). By January 2017, 1462 patients with axSpA who started TNFi treatment were consecutively enrolled from 47 tertiary referral centers in South Korea. A patient was enrolled when he/she started a new TNFi treatment and was followed up annually. If a patient stopped the TNFi treatment, the reason and date of discontinuation were recorded. Because KOBIO cohort is an observational cohort, treatment decision was made by patients and their physicians. However, all the patients started TNFi treatment at the standard dose. For data quality control, queries regarding incomplete data were regularly sent to each hospital for clarification.

In this study, we excluded patients who missed follow-up visits (*n* = 363) or those with missing information on the TNFi dose (*n* = 15). In addition, patients treated with TNFi for less than 1 year (*n* = 198) were excluded because it is less likely that they achieved the prolonged stable disease activity required to be eligible for tapering TNFi in real-world settings. In fact, patients in the KOBIO cohort discontinued TNFi treatment due to inefficacy (*n* = 79), adverse drug reactions (*n* = 55), or poor compliance/lost to follow-up (*n* = 40), supporting our clinical assumption. A flow chart depicting patient inclusion is presented in online Additional file 1: Figure S1.

The study was carried out in accordance with the Declaration of Helsinki and was approved by the institutional review boards of all participating hospitals. Written informed consent was obtained from all the patients.

### Data collection

At the time of enrolment (defined as the baseline visit), the baseline data of patients regarding demographics, body mass index, smoking status (ever vs. never), previous treatment, HLA-B27 positivity, presence of definite sacroiliitis (defined as bilateral sacroiliitis ≥ grade 2 or unilateral sacroiliitis ≥ grade 3) in plain radiographs, and baseline disease activity indices such as Ankylosing Spondylitis Disease Activity Score (ASDAS), Bath Ankylosing Spondylitis Disease Activity Index (BASDAI), and Bath Ankylosing Spondylitis Functional Index (BASFI) were collected. At each annual follow-up visit, the data on disease activity indices, concomitant medications (non-steroidal anti-inflammatory drugs and methotrexate), and cumulative dose of TNFi were collected. The TNFi dose during 1-year follow-up interval was quantified as dose quotient (DQ), calculated as (mean actual dose/standard dose) × (standard − dosing interval/mean actual dosing interval) × 100 [12]. The observation period of this study was 3 years from the baseline visit or the time to the discontinuation of TNFi starting at baseline, whichever was first.

### Change in DQ and outcomes

Because there is no universal recommendation regarding tapering of TNFi in patients with axSpA, DQ of the 1-year follow-up interval continuously changed during the observation period (Additional file 1: Figure S2). In addition, DQ could be influenced by prior disease activity in real-world settings, and it was true for our data (*P* value in type 3 test of fixed effect was < 0.001). This suggests that assessments on TNFi tapering at the individual level cannot precisely estimate the effect of tapering on the activity of axSpA. Therefore, we performed a longitudinal analysis where each 1-year interval from all the included patients was used as an observational unit. All 1-year intervals were classified into one of the following three groups according to their DQ (< 50, heavy-tapering group; 50–99, mild-tapering group; and 100, control group).

The primary outcome was achieving ASDAS-inactive disease (ASDAS-ID, defined as ASDAS-CRP < 1.3) at the follow-up visit. The secondary outcome included achieving BASDAI50 response criteria. In addition, the impact of DQ on the likelihoods of achieving ASAS20/40, ASDAS-low disease activity (ASDAS-LDA, defined as 1.3 ≤ ASDAS-CRP < 2.1), BASDAI < 4, and C-reactive protein (CRP) < 0.5 mg/dL at the follow-up visit was also investigated.

### Statistical analysis

All statistical analyses were based on observational data, and missing data were not imputated. The relationship between dosing strategy of TNFi and longitudinal disease activity was analyzed using generalized estimating equations (GEEs) considering repeated measurements for each patient [17]. An “exchangeable” correlation matrix was selected based on the correlation coefficients among the outcomes at each interval. Univariable GEE was used to determine the clinical factors associated with the outcomes in the 1-year interval. If a factor showed a relevant association with the outcome (*P* < 0.2), it was included as a covariate in the multivariable model.

We constructed two different multivariable models. The first model (baseline model) included only covariates measured at the baseline visit. The second model (longitudinal model) included relevant covariates among consecutively measured clinical factors such as disease activity at each follow-up visit and concomitant medication during the 1-year follow-up interval. In addition, because the effect of prior disease activity could differ depending on the group, interaction between the group and prior disease activity was also included if it was relevant to the outcome in the univariable GEE. The fitness of the model was assessed using quasi-likelihood under the independence model criterion (QIC). For the sensitivity analysis, we constructed another multivariable model where all covariates with a clinically relevant association with ASDAS-ID achievement were included. In addition, the same GEE was used in the subgroup of patients for whom complete follow-up data were available (*n* = 227). All statistical analyses were performed using SPSS 20.0 software (Armonk, NY: IBM Corp.). The results with a *P* value < 0.05 were considered statistically significant.

## Results

### Patient characteristics

We analyzed 1575 1-year follow-up interval data of 776 patients. The number of intervals in the control, mild-tapering, and heavy-tapering groups was 1091 (69.3%), 440 (27.9%), and 44 (2.8%), respectively. All patients treated with subcutaneous agents (etanercept, adalimumab, and golimumab) tapered their TNFi by prolonging the dosing interval. Among the 176 intervals with tapered infliximab treatment, 120 (68.2%) intervals reduced the infusion dose and 67 (38.1%) increased the dosing interval. Methotrexate (MTX) and sulfasalazine were concomitantly administered in 38 (2.4%) and 21 (1.3%) intervals, respectively.

Throughout the observation period, 77 (9.9%) patients stopped the TNFi treatment. Among them, 21 (2.7%) and 16 (2.1%) patients discontinued the treatment due to inefficacy and adverse events, respectively.

The baseline characteristics of the patients are summarized in Table 1. Briefly, the mean (SD) age of patients was 37.8 (12.5) years; 78% of them were male. More than 90% of the patients were HLA-B27 positive. Approximately 90% of the patients (681/776, 87.8%) showed definite radiographic sacroiliitis in plain radiographs and fulfilled the modified New York criteria for ankylosing spondylitis (AS) [18]. The mean (SD) ASDAS-CRP at baseline was 3.6 (1.1), and most patients (91.1%) showed a high disease activity (ASDAS-CRP ≥ 2.1).

**Table 1:** Baseline features of the patients in the KOBIO cohort at the start of TNFi treatment

Clinical feature	*N* = 776
Age, years, mean (SD)	37.8 (12.5)
Male gender, *n* (%)	605 (78.0)
Disease duration, years, mean (SD)	8.1 (6.0)
BMI, mean (SD)	23.4 (3.5)
Obesity, *n* (%)	228 (29.4)
Ever-smoker, *n* (%)	385 (49.6)
HLA-B27 positive, *n* (%)*	696 (90.7)
Ankylosing spondylitis, *n* (%)	681 (87.8)
TNFi naïve, *n* (%)	608 (78.4)
TNFi agent	
Infliximab	201 (25.9)
Etanercept	104 (13.4)
Adalimumab	282 (36.3)
Golimumab	189 (24.4)
ESR, mm/h, mean (SD)^†^	38.3 (30.5)
CRP, mg/dL, mean (SD)^‡^	2.3 (2.8)
PGA (0–10), mean (SD)	6.2 (2.2)
BASDAI (0–10), mean (SD)	6.0 (1.9)
ASDAS-CRP, mean (SD)	3.6 (1.1)
BASFI, mean (SD)	3.5 (2.6)

*AS* ankylosing spondylitis, *ASDAS* Ankylosing Spondylitis Disease Activity Score, *BASDAI* Bath Ankylosing Spondylitis Disease Activity Index, *BASFI* Bath Ankylosing Spondylitis Functional Index, *BMI* body mass index, *CRP* C-reactive protein, *ESR* erythrocyte sedimentation rate, *HLA* human leukocyte antigen, *PGA* patient global assessment, *SD* standard deviation

*There were 9 missing data

^†^There were 16 missing data

^‡^There were 19 missing data

### Likelihood of outcome achievement during the follow-up

**Fig. 1:**
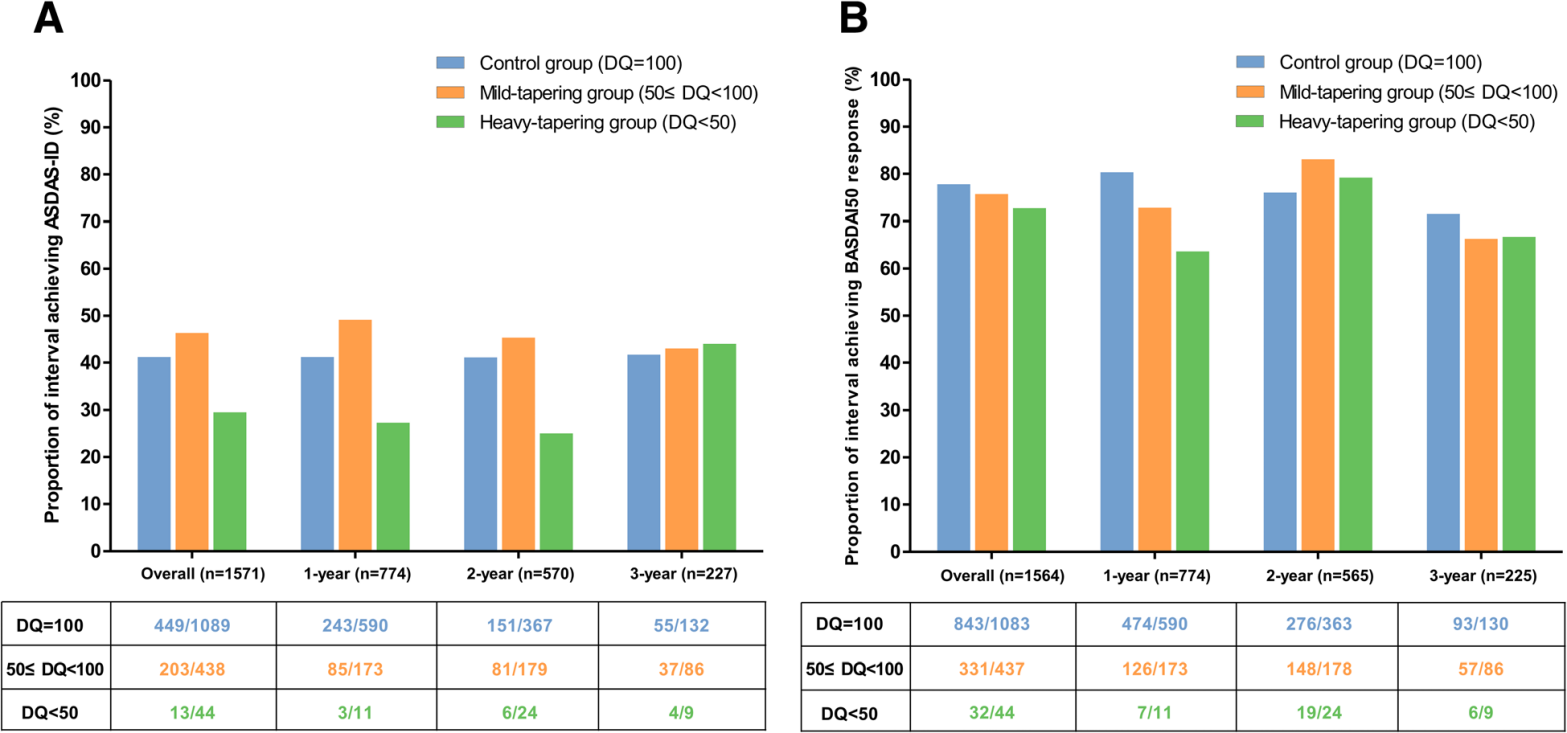
Likelihood of achieving **a** ASDAS-inactive disease and **b** BASDAI50 response in the 1-year interval among the three groups during observation. ASDAS, Ankylosing Spondylitis Disease Activity Score; axSpA, axial spondyloarthritis; BASDAI, Bath Ankylosing Spondylitis Disease Activity Index

During the observation period, ASDAS-ID was observed in 665 (42.3%) intervals. This proportion did not change with the follow-up year. The likelihood of primary outcome achievement was comparable between the control and mild-tapering groups (41.2% and 46.3%, respectively), but was markedly lower in the heavy-tapering group (29.5%) (Fig. 1a). In contrast, the BASDAI50 response criterion was fulfilled in a significantly higher number of intervals than ASDAS-ID was (77.1%). It remained unchanged with the follow-up time and was comparable among the three groups (Fig. 1b).

### Tapering of TNFi and achieving ASDAS-ID

The analysis using univariable GEE showed that older age, obesity, ever-smoking status, negative HLA-B27, and definite sacroiliitis in the baseline radiographs were associated with a lower possibility of achieving ASDAS-ID during the treatment. A higher ASDAS-CRP at the baseline also decreased the likelihood of reaching ASDAS-ID. An increase in one unit of baseline ASDAS-CRP reduced the odds by 23% (OR = 0.77 [95% confidence interval (CI), 0.69–0.87]). Among the longitudinal factors, the likelihood of achieving ASDAS-ID was significantly influenced by ASDAS-CRP in the prior follow-up visit (ASDAS_*t* − 1_) (OR = 0.92 [95% CI, 0.86–0.98]). Concomitant NSAID and sulfasalazine uses during the follow-up interval were also associated with a lower probability of achieving ASDAS-ID.

The analysis with multivariable GEE showed a comparable likelihood of reaching the target in the control and mild-tapering groups. However, the likelihood was significantly lower in the heavy-tapering group compared with the control group. Both models showed consistent results, but the QIC was relatively lower in the longitudinal model (Table 2). We also performed the longitudinal GEE model in which the difference of ASDAS-CRP was used as an outcome variable. In this analysis, reduction in ASDAS-CRP compared with its baseline value in the control group was comparable to that in the mild-tapering group, but was significantly greater than the reduction in the heavy-tapering group (adjusted *β* = 0.45, 95% CI 0.06–0.83).

**Table 2:** Effect of tapering TNFi on the achievement of consecutive ASDAS-ID in the 1-year interval

	Univariable model, OR (95% CI) (*n* = 757–776)	Baseline model*OR (95% CI) (*n* = 767)	Longitudinal model^⁋^OR (95% CI) (*n* = 767)
Baseline variable			
Age, 10 years	0.87 (0.79–0.97)	0.91 (0.82–1.01)	0.92 (0.83–1.02)
Female sex	0.98 (0.73–1.30)	‡	‡
Disease duration, 10 years	0.81 (0.66–1.002)	0.81 (0.65–1.02)	0.78 (0.61–0.98)
Obesity (BMI ≥ 25)	0.71 (0.54–0.93)	0.78 (0.59–1.04)	0.77 (0.58–1.01)
Ever-smokers	0.73 (0.57–0.93)	0.81 (0.63–1.04)	0.83 (0.64–1.08)
Positive HLA-B27 (vs. negative)	1.63 (1.03–2.56)	1.72 (1.09–2.71)	1.71 (1.08–2.73)
AS (vs. nr-axSpA)	0.66 (0.45–0.96)	0.71 (0.48–1.01)	0.72 (0.49–1.06)
TNFi naïve	0.98 (0.73–1.31)	‡	‡
Baseline ASDAS-CRP, unit	0.77 (0.69–0.87)	0.76 (0.68–0.86)	0.78 (0.69–0.89)
Longitudinal variable			
Follow-up time (vs. interval in the 1-year follow-up)	Reference	†	‡
Interval in the 2-year follow-up	0.98 (0.82–1.17)	†	‡
Interval in the 3-year follow-up	1.06 (0.83–1.36)	†	‡
ASDAS-CRP_*t* − 1_, unit	0.92 (0.86–0.98)	†	0.95 (0.86–1.05)
Concomitant NSAID use during the interval	0.44 (0.35–0.56)	†	0.45 (0.35–0.58)
Concomitant sulfasalazine use during the interval	0.31 (0.11–0.94)	†	0.29 (0.09–0.90)
Concomitant MTX use during the interval	1.37 (0.76–2.44)	†	‡
Group according to the interval DQ			
Control group (DQ = 100)	Reference	Reference	Reference
Mild-tapering group (50 ≤ DQ < 100)	1.21 (0.98–1.48)	1.19 (0.96–1.48)	0.89 (0.56–1.41)
Heavy-tapering group (DQ < 50)	0.44 (0.22–0.88)	0.43 (0.22–0.85)	0.27 (0.08–0.91)
QIC of the model		2059.054	2002.264

*AS* Ankylosing spondylitis, *ASDAS* Ankylosing Spondylitis Disease Activity Score, *BMI* body mass index, *CI* confidence interval, *CRP* C-reactive protein, *DQ* dose quotient, *HLA* human leukocyte antigen, *MTX* methotrexate, *nr-axSpA* non-radiographic axial spondyloarthritis, *NSAID* non-steroidal anti-inflammatory drug, *OR* odds ratio, *QIC* quasi-likelihood under the independence model criterion, *TNFi* tumor necrosis factor inhibitor

*The model was adjusted for the baseline clinical factors showing a relevant association (*P* < 0.2) with the outcome in the univariable model

^†^Not included in the model

^‡^Not included in the model because its association with the outcome was not relevant (*P* ≥ 0.2)

^⁋^The model was adjusted for covariates in the baseline model and longitudinal factors with a relevant association (*P* < 0.2) with the outcome in the univariable model

### Tapering of TNFi and fulfilling of BASDAI50 criteria

BASDAI50 response was achieved in 1207 (77.1%) of the whole intervals. The analysis results of univariable GEE in which BASDAI50 criterion fulfillment was used as an outcome are presented in Table 3. Briefly, obesity, smoking, and the presence of definite sacroiliitis in radiographs, which impair the treatment response of TNFi, were not associated with the outcome. On the contrary, the criteria were more likely to be fulfilled among TNFi-naïve patients. Of note, unlike the ASDAS-ID, there was no significant difference in the likelihood of fulfilling the BASDAI50 criteria among the three groups.

**Table 3:** Effect of tapering TNFi on the achievement of consecutive BASDAI50 response in the 1-year interval

	Univariable modelOR (95% CI) (*n* = 757–776)	Baseline model*OR (95% CI) (*n* = 748)	Longitudinal model^⁋^OR (95% CI) (*n* = 748)
Baseline variable			
Age, 10 years	0.81 (0.72–0.91)	0.83 (0.72–0.95)	0.83 (0.73–0.94)
Female sex	0.80 (0.56–1.13)	‡	‡
Disease duration, 10 years	0.69 (0.55–0.85)	0.89 (0.67–1.17)	0.89 (0.68–1.17)
Obesity (BMI ≥ 25)	0.88 (0.64–1.20)	‡	‡
Ever-smokers	0.89 (0.67–1.19)	‡	‡
Positive HLA-B27 (vs. negative)	1.69 (1.04–2.74)	1.50 (0.83–2.71)	1.47 (0.84–2.56)
AS (vs. nr-axSpA)	0.77 (0.48–1.21)	‡	‡
TNFi naïve	1.75 (1.26–2.43)	1.49 (1.01–2.20)	1.44 (0.98–2.10)
Baseline BASDAI, unit	1.64 (1.52–1.78)	1.61 (1.48–1.75)	1.93 (1.72–2.16)
Baseline CRP, mg/dL	1.21 (1.10–1.33)	1.13 (1.04–1.23)	1.13 (1.04–1.22)
Longitudinal variable			
Follow-up time (vs. interval in the 1-year follow-up)	Reference	†	Reference
Interval in the 2-year follow-up	0.99 (0.81–1.20)	†	0.52 (0.35–0.77)
Interval in the 3-year follow-up	0.60 (0.46–0.79)	†	0.27 (0.16–0.45)
BASDAI_*t* − 1_, unit	1.12 (1.08–1.15)	†	0.78 (0.70–0.89)
CRP_*t* − 1_, mg/dL	1.11 (1.04–1.18)	†	1.01 (0.94–1.08)
Concomitant NSAID use during the interval	0.80 (0.60–1.07)	†	‡
Concomitant sulfasalazine use during the interval	0.69 (0.32–1.47)	†	‡
Concomitant MTX use during the interval	1.04 (0.49–2.24)	†	‡
Group according to the interval DQ			
Control group (DQ = 100)	Reference	Reference	Reference
Mild-tapering group (50 ≤ DQ < 100)	0.93 (0.73–1.20)	0.94 (0.70–1.26)	1.03 (0.61–1.74)
Heavy-tapering group (DQ < 50)	0.80 (0.43–1.51)	0.82 (0.34–1.97)	1.09 (0.26–4.55)
QIC of the model		1367.597	1317.502

*AS* ankylosing spondylitis, *BASDAI* Bath Ankylosing Spondylitis Activity Index, *BMI* body mass index, *CI* confidence interval, *CRP* C-reactive protein, *DQ* dose quotient, *HLA* human leukocyte antigen, *MTX* methotrexate, *nr-axSpA* non-radiographic axial spondyloarthritis, *NSAID* non-steroidal anti-inflammatory drug, *OR* odds ratio, *QIC* quasi-likelihood under the independence model criterion, *TNFi* tumor necrosis factor inhibitor

*The model was adjusted for baseline clinical factors showing a relevant association (*P* < 0.2) with the outcome in the univariable model

^†^Not included in the model

^‡^Not included in the model because its association with the outcome was not relevant (*P* ≥ 0.2)

^⁋^The model was adjusted for covariates in the baseline model and longitudinal factors with a relevant association (*P* < 0.2) with the outcome in the univariable model

### Tapering of TNFi and achieving other clinical outcomes

Proportion of 1-year intervals which achieved other clinical outcomes such as ASDAS-LDA, ASAS20 and 40, BASDAI < 4, and CRP < 0.5 mg/dL during the observation is presented in Additional file 1: Figure S3. Briefly, ASDAS-LDA was achieved in 512 (32.6%) 1-year intervals and the likelihood for the target was not significantly different among the three groups (33.2% in the control group, 30.4% in the mild-tapering group, and 38.6% in the heavy-tapering group). This result was not changed in the multivariable GEE model where concomitant NSAID and sulfasalazine uses were adjusted (Additional file 1: Table S1).

ASAS20 and 40 were achieved in 1141 (75.2%) and 1011 (66.6%) of the entire 1-year intervals, respectively. In the multivariable analyses, likelihoods of achieving ASAS20 and 40 were comparable between the control and mild-tapering groups, but were significantly lower in the heavy-tapering group. This result was consistent in another multivariable GEE model where BASDAI < 4 and CRP < 0.5 mg/dL were selected as the outcome (Additional file 1: Table S1).

### Effect of TNFi tapering according to the disease activity at the 1-year follow-up visit

**Fig. 2:**
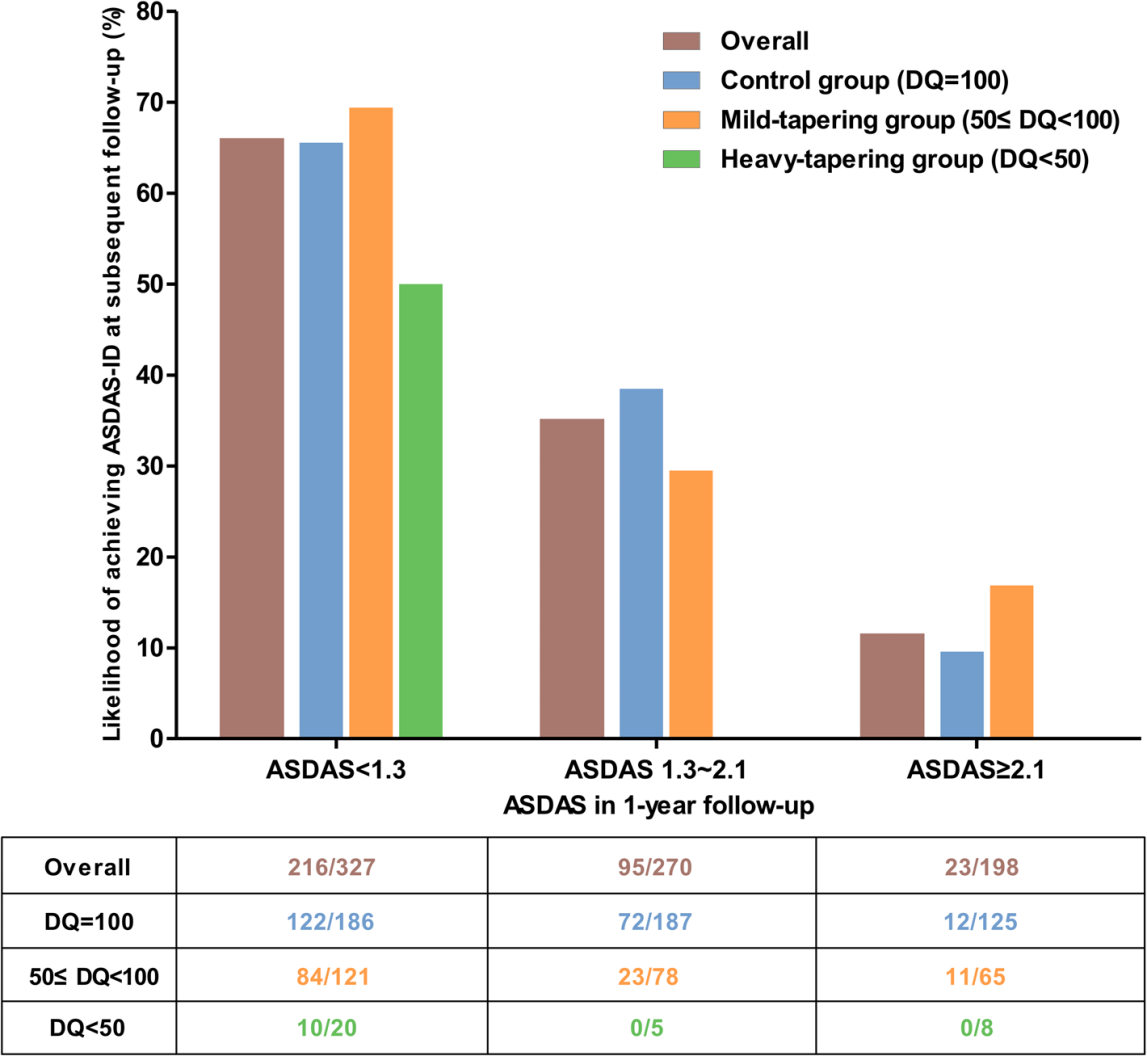
Likelihood of achieving ASDAS-inactive disease in the subsequent intervals according to the ASDAS-CRP measured at the 1-year follow-up. ASDAS, Ankylosing Spondylitis Disease Activity Score; axSpA, axial spondyloarthritis; CRP, C-reactive protein

As the likelihood of ASDAS-ID achievement in the 1-year interval was low, we stratified all patients by the ASDAS-CRP measured at the 1-year follow-up visit and analyzed the probability of achieving ASDAS-ID at the subsequent visits in each subgroup. Interestingly, this probability increased as the ASDAS-CRP at the 1-year follow-up decreased (Fig. 2). In the subgroup of patients who achieved ASDAS-ID at the 1-year follow-up (*n* = 327), the target was successfully maintained in 66.1% of the subsequent intervals. Multivariable analysis in this population showed that the mild-tapering group, but not the heavy-tapering group, had a likelihood of retaining the inactive disease state comparable to that of the control group (Table 4). In contrast, among patients who achieved ASDAS-LDA (1.3 ≤ ASDAS-CRP < 2.1) (*n* = 254), the probability of achieving ASDAS-ID at the subsequent intervals was only 35.2%. Moreover, tapering of TNFi in this subgroup further decreased the likelihood, irrespective of the extent of tapering in the multivariable longitudinal model (adjusted OR = 0.53 [95% CI, 0.29–0.97]) (Additional file 1: Table S2). In the last subgroup with ASDAS-CRP higher than 2.1 at the 1-year follow-up, only 11.6% (23/198) patients subsequently achieved the target.

**Table 4:** Effect of tapering DQ on maintaining ASDAS-ID in patients who achieved ASDAS-ID at 1-year follow-up

	Number (%) of 1-year intervals maintained the ASDAS-ID	Univariable modelOR (95% CI) (*n* = 230–236)	Baseline model* OR (95% CI) (*n* = 236)	Longitudinal model^†^ OR (95% CI) (*n* = 236)
Group according to the interval DQ				
Control group (DQ = 100)	122 (65.6)	Reference	Reference	Reference
Mild-tapering group (50 ≤ DQ < 100)	84 (69.4)	1.16 (0.70–1.45)	1.22 (0.73–2.04)	1.25 (0.41–3.80)
Heavy-tapering group (DQ < 50)	10 (50.0)	0.58 (0.23–1.45)	0.57 (0.23–1.41)	0.19 (0.05–0.74)
QIC of the model		422.244	413.739	407.520

*ASDAS* Ankylosing Spondylitis Disease Activity Score, *CI* confidence interval, *DQ* dose quotient, *OR* odds ratio, *QIC* quasi-likelihood under the independence model criterion

*The model was adjusted for age, sex, and baseline ASDAS-CRP

^†^The model was adjusted for covariates in the baseline model, ASDAS_*t* − 1_, concomitant NSAID, and interaction between ASDAS_*t* − 1_ and group

### Sensitivity analysis

In the multivariable model in which all factors with clinical relevance to the outcome were included, the result was consistent with that drawn from the main analysis; likelihood for achieving ASDAS-ID was comparable between the control and the mild-tapering groups but was significantly lower in the heavy-tapering group (Additional file 1: Table S3). In addition, the subgroup analysis including patients who had completed a 3-year observation period (*n* = 271) showed that ASDAS-ID was achieved in 40.2% (274/681) of the intervals, which was comparable to that of the entire population. The multivariable model also showed consistent results. The likelihood of achieving ASDAS-ID was not significantly different between the control and mild-tapering groups, but tended to decrease in the heavy-tapering group (Additional file 1: Table S4).

## Discussion

Although tapering of TNFi was first introduced in the recent ASAS/EULAR guideline, its efficacy compared with that of the standard-dose treatment has not been thoroughly evaluated. Previous studies that investigated the efficacy of the tapering strategy included a small number of patients and had a relatively short observation period [11–14, 19]. Furthermore, indications for tapering and the definition of clinical outcomes in these studies were heterogeneous, making it difficult to interpret the results. To the best of our knowledge, this is the first large-scale study to investigate the efficacy of tapering TNFi in real-world clinical settings, from the perspective of achieving ASDAS-ID as the optimal treatment target.

In this study, ASDAS-ID was achieved in only 42.3% of the entire 1-year intervals. Considering that patients with TNFi treatment for less than 1 year were excluded, the possibility of achieving the target could be lower in real-world settings [20, 21]. The finding is also consistent with the results of previous randomized controlled trials where the probability of achieving ASDAS-ID with TNFi treatment ranged between 16.1 and 36.5% [5, 22–24]. In contrast, BASDAI50 criteria were fulfilled in approximately 80% of the intervals, indicating that it is a more lenient target than ASDAS-ID. Interestingly, only a small number of patients discontinued the TNFi treatment due to loss of efficacy judged by their physician. This result suggests that continuing TNFi treatment in patients with axSpA is more influenced by a patient’s subjective symptoms rather than stringent current guidelines in real-world settings. It can also be attributed to the fact that other bDMARDs such as IL-17A inhibitors were not available in South Korea during the study period. In addition, because the second or third TNFi usually shows a lower response rate than that by the first one, physicians might have hesitated to change the treatment if a patient had marginally high ASDAS with relatively mild symptoms [25]. Therefore, more evidence regarding the efficacy of alternative therapeutic options after the failure of TNFi treatment is required for the universal recommendation of the “treat-to-target” strategy.

Our longitudinal analysis also demonstrated that the standard dose of TNFi and mild tapering of TNFi were associated with a comparable likelihood of achieving ASDAS-ID in the 1-year interval, but the likelihood significantly decreased in the heavy-tapering group. In contrast, the likelihood of fulfilling the BASDAI50 criteria was not different among the three groups. Other clinical outcomes such as ASAS20/40 and CRP < 0.5 mg/dL showed the same trend with ASDAS-ID. Since ASAS20/40, CRP, and ASDAS-CRP put more weight on inflammation than BASDAI, it is likely that the discrepancy between the ASDAS-ID and BASDAI50 was derived from the difference in inflammation control between the control and heavy-tapering group. This discrepancy again suggests that a patient’s subjective symptom alone cannot precisely estimate the activity of axSpA [26]. The results indicate that caution must be exerted while tapering TNFi by more than 50% of the standard dose even if a patient’s symptoms are well controlled. However, because the number of 1-year intervals in the heavy-tapering group was small, this result should be confirmed in the future.

The relatively low likelihood of achieving ASDAS-ID in the follow-up intervals also suggests that non-selective application of TNFi tapering in patients receiving TNFi treatment for more than 1 year is not appropriate to reach the optimal goal. We found that patients who achieved ASDAS-ID at the 1-year follow-up were highly likely to maintain the target (66.1%) and that mild tapering of TNFi did not influence this. It implies that achieving ASDAS-ID after 1 year of TNFi treatment could be an appropriate indication to consider tapering of TNFi. However, even in this subgroup, heavy tapering of TNFi was associated with a low likelihood of maintaining the goal.

The limitations of this study include the following. First, because it is an observational study, the results could have been biased due to confounding by indication. For example, data on the reason for tapering TNFi is lacking, so it is uncertain whether it was performed strictly based on the patient’s disease activity. We demonstrated that tapering and its extent were considered to be at least partially guided by patients’ disease activity. However, although this interaction was adjusted in the longitudinal model, some unmeasured confounders such as patient’s compliance and economic status and physician preference for tapering TNFi cannot be completely balanced. Regarding the economic burden, however, all AS patients in South Korea copay 10% of the cost of all prescribed medication (including TNFi) under the coverage of national health insurance system [27]. Therefore, it is less likely that the decision on tapering TNFi was driven by the factor. Second, this study did not show the effect of tapering TNFi on maintaining ASDAS-ID for more than 1 year in patients because the observational unit of this study was not an individual patient. To clarify this important aspect, a randomized controlled trial with a well-designed protocol will be required. Third, because KOBIO being a recently established ongoing registry, there was relatively a small number of patients, especially in the heavy-tapering group, who completed the 3-year follow-up visit. Finally, the incidence of adverse drug reactions related to TNFi and its relationship to tapering were not thoroughly investigated in this study.

## Conclusions

In summary, our study showed that mild tapering of TNFi had efficacy comparable with that of the standard-dose treatment in maintaining the optimal target in patients with axSpA who reached ASDAS-ID 1 year after TNFi treatment. Although this result should be confirmed with randomized studies in the future, it provides important real-world evidence for universal recommendation of the tapering strategy.

## Additional file

Additional file 1: **Figure S1.** Flow chart of inclusion. **Figure S2.** Dynamic changes in dose quotient (DQ) of TNFi in included patients during the follow-up (time level). **Figure S3.** Proportion of 1-year intervals achieving (A) ASDAS-low disease activity, (B) ASAS20, (C) ASAS40, (D) BASDAI < 4, and (E) CRP < 0.5 mg/dL. **Table S1.** Effect of tapering TNFi on the achievement of various outcomes in 1-year intervals. **Table S2.** Effect of tapering DQ on maintaining ASDAS-ID in subsequent 1-year intervals in the subgroup of patients who showed ASDAS-low disease activity (1.3 ≤ ASDAS-CRP < 2.1) at 1-year follow-up (*n* = 254). **Table S3.** Multivariable longitudinal model where all clinically relevant factors were included as covariates. **Table S4.** Effect of tapered DQ on the achievement of consecutive ASDAS-ID in the subgroup of patients who completed 3-year follow-up. (DOCX 1150 kb)

## Data Availability

All of the data supporting the conclusions of this article are included within the article.

## Abbreviations

ASAS: Assessment of SpondyloArthritis International Society
ASDAS: Ankylosing Spondylitis Disease Activity Score
axSpA: Axial spondyloarthritis
BASDAI: Bath Ankylosing Spondylitis Disease Activity Index
BASFI: Bath Ankylosing Spondylitis Functional Index
CRP: C-reactive protein
DMARD: Disease-modifying antirheumatic drug
DQ: Dose quotient
GEE: Generalized estimating equation
HLA: Human leukocyte antigen
ID: Inactive disease
IL: Interleukin
NSAID: Non-steroidal anti-inflammatory drug
SD: Standard deviation
TNFi: Tumor necrosis factor inhibitor
