# Supplementary material for: Effects of tapering tumor necrosis factor inhibitor on the achievement of inactive disease in patients with axial spondyloarthritis: a nationwide cohort study
Source: Arthritis Res Ther. 2019 Jul 4;21:163. doi: 10.1186/s13075-019-1943-6 (PMC6611048; doi:10.1186/s13075-019-1943-6)
Supplement: Supplementary file 1 — Figure S1. Flow chart of inclusion. Figure S2. Dynamic changes in dose quotient (DQ) of TNFi in included patients during the follow-up (time level). Figure S3. Proportion of 1-year intervals achieving (A) ASDAS-low disease activity, (B) ASAS20, (C) ASAS40, (D) BASDAI < 4, and (E) CRP < 0.5 mg/dL. Table S1. Effect of tapering TNFi on the achievement of various outcomes in 1-year intervals. Table S2. Effect of tapering DQ on maintaining ASDAS-ID in subsequent 1-year intervals in the subgroup of patients who showed ASDAS-low disease activity (1.3 ≤ ASDAS-CRP < 2.1) at 1-year follow-up (n = 254). Table S3. Multivariable longitudinal model where all clinically relevant factors were included as covariates. Table S4. Effect of tapered DQ on the achievement of consecutive ASDAS-ID in the subgroup of patients who completed 3-year follow-up. (DOCX 1150 kb) [file 13075_2019_1943_MOESM1_ESM.docx]

**Supplementary figure S1.** Flow chart of inclusion

**Supplementary figure S2.** Dynamic changes in dose quotient (DQ) of TNFi in included patients during the follow up (time level)

**Supplementary figure S3.** Proportion of one-year intervals achieving (A) ASDAS-low disease activity, (B) ASAS20, (C) ASAS40, (D) BASDAI < 4, and (E) CRP < 0.5mg/dL
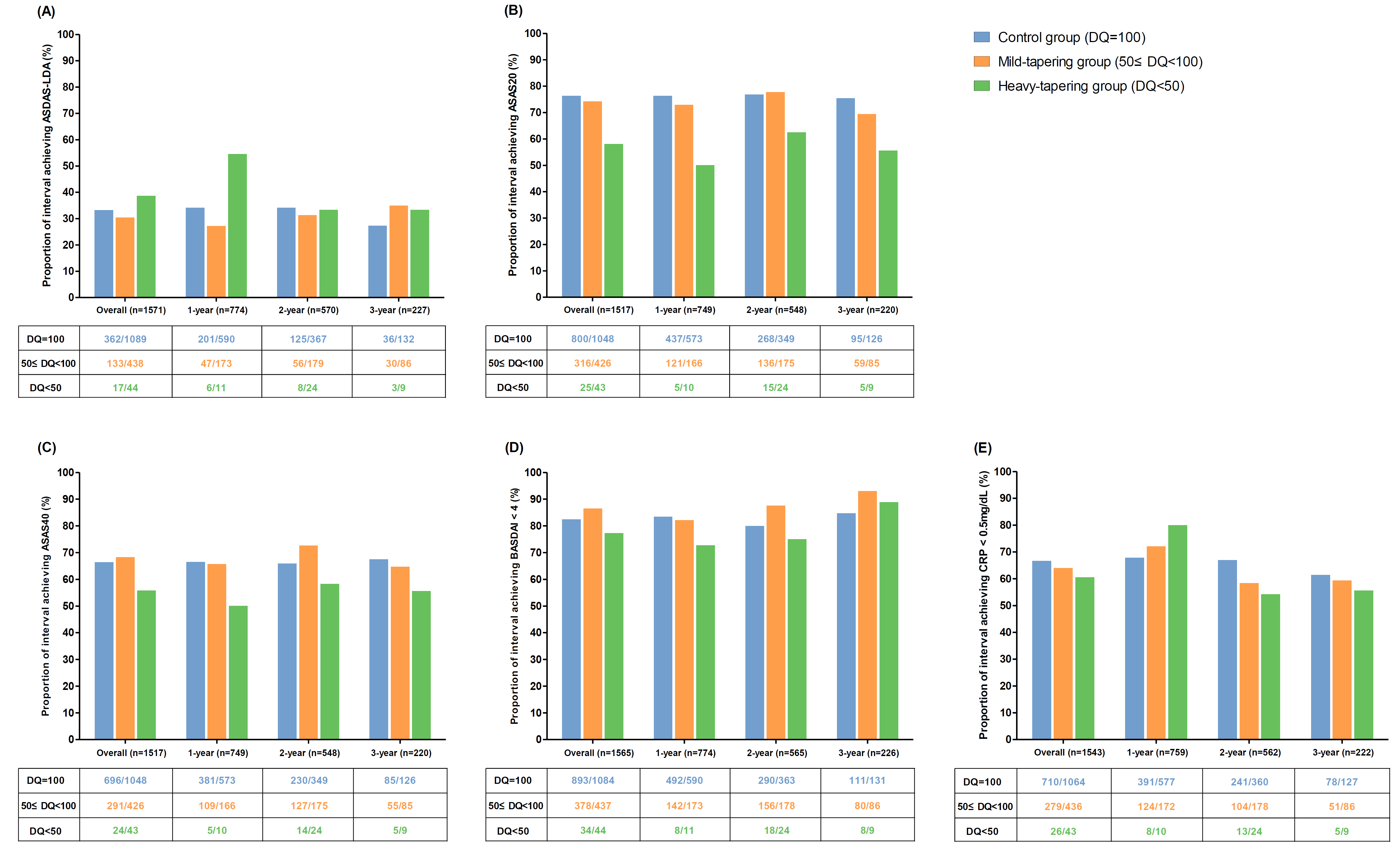


**Supplementary table S1.** Effect of tapering TNFi on the achievement of various outcomes in one-year intervals

| Outcome variable | Univariable model  OR (95% CI) | Multivariable model^*^  OR (95% CI) |
| --- | --- | --- |
| ASDAS-LDA (n = 776) |  |  |
| Control group (DQ = 100) | Reference | Reference |
| Mild-tapering group (50 ≤ DQ < 100) | 0.91 (0.72–1.16) | 0.93 (0.73–1.19) ^†^ |
| Heavy-tapering group (DQ < 50) | 1.39 (0.77–2.53) | 1.46 (0.81–2.65) ^†^ |
| ASAS20 response (n = 754) |  |  |
| Control group (DQ = 100) | Reference | Reference |
| Mild-tapering group (50 ≤ DQ < 100) | 0.92 (0.72–1.16) | 0.86 (0.66–1.13) ^‡^ |
| Heavy-tapering group (DQ < 50) | 0.45 (0.23–0.89) | 0.31 (0.12–0.78) ^‡^ |
| ASAS40 response (n = 754) |  |  |
| Control group (DQ = 100) | Reference | Reference |
| Mild-tapering group (50 ≤ DQ < 100) | 1.17 (0.93–1.48) | 1.07 (0.83–1.40) ^⁋^ |
| Heavy-tapering group (DQ < 50) | 0.61 (0.31–1.20) | 0.44 (0.17–1.14) ^⁋^ |
| BASDAI < 4 (n = 748) |  |  |
| Control group (DQ = 100) | Reference | Reference |
| Mild-tapering group (50 ≤ DQ < 100) | 1.48 (1.09–2.02) | 1.27 (0.90–1.80)^∮^ |
| Heavy-tapering group (DQ < 50) | 0.66 (0.33–1.31) | 0.45 (0.21–0.99)^∮^ |
| CRP < 0.5mg/dL (n = 753) |  |  |
| Control group (DQ = 100) | Reference | Reference |
| Mild-tapering group (50 ≤ DQ < 100) | 0.96 (0.79–1.15) | 1.02 (0.83–1.25) ^∫^ |
| Heavy-tapering group (DQ < 50) | 0.60 (0.36–1.03) | 0.68 (0.39–1.17) ^∫^ |

ASDAS, Ankylosing Spondylitis Disease Activity Score; BASDAI, Bath Ankylosing Spondylitis Activity Index; CI, confidence interval; DQ, dose quotient; HLA, human leukocyte antigen; NSAID, nonsteroidal anti-inflammatory drug; OR, odds ratio.

^*^, The model was adjusted for baseline and longitudinal factors with a relevant association (P < 0.2) with the outcome in the univariable model.

^†^, was adjusted for concomitant NSAID and sulfasalazine use.

^‡^, was adjusted for age, disease duration, TNFi naïve, HLA-B27, baseline ASDAS-CRP, ASDAS-CRP _t-1_ and concomitant NSAID use.

^⁋^, was adjusted for age, disease duration, obesity, definite sacroiliitis, TNFi naïve, baseline ASDAS-CRP, ASDAS-CRP _t-1_ and concomitant NSAID use.

^∮^, was adjusted for age, sex, obesity, HLA-B27, TNFi naïve, follow-up year, baseline BASDI and CRP, BASDAI_t-1_, and concomitant NSAID use.

^∫^, was adjusted for age, sex, obesity, ever smoking, definite radiographic sacroiliitis, follow-up year, TNFi naïve, baseline CRP and concomitant NSAID use.

**Supplementary table S2.** Effect of tapering DQ on maintaining ASDAS-ID in subsequent one-year intervals in the subgroup of patients who showed ASDAS-low disease activity (1.3 ≤ ASDAS-CRP < 2.1) at 1-year follow-up (n = 254)

|  | Number (%) of 1-year intervals maintained the ASDAS-ID | Univariable model  OR (95% CI)  (n = 254) | Longitudinal model^†^  OR (95% CI)  (n = 254) |
| --- | --- | --- | --- |
| Group according to the interval DQ |  |  |  |
| Control group (DQ=100) | 72 (38.5) | Reference | Reference |
| Tapering group (DQ<100)^*^ | 23 (27.7) | 0.90 (0.53–1.52) | 0.53 (0.28–0.98) |
| QIC of the model |  | 499.892 | 371.438 |

ASDAS, Ankylosing Spondylitis Disease Activity Score; CI, confidence interval; DQ, dose quotient; OR, odds ratio; QIC, Quasi-likelihood under the Independence model Criterion.

^*^, Stratification of the group based on DQ=50 was not available due to the small number of heavy-tapering group (n=11).

^†^, The model was adjusted for ASDAS_t-1_ and concomitant NSAID. No baseline clinical factors showed a relevant association with the outcome in the univariable analysis.

**Supplementary table S3** Multivariable longitudinal model where all clinically relevant factors were included as covariates

|  | Univariable model  OR (95% CI)  (n = 757–776) | Multivariable model^*^  OR (95% CI)  (n = 767) |
| --- | --- | --- |
| Baseline variable |  |  |
| Age, 10 years | 0.87 (0.79–0.97) | 0.94 (0.84–1.04) |
| Female sex | 0.98 (0.73–1.30) | 0.70 (0.50–0.99) |
| Disease duration, 10 years | 0.81 (0.66–1.002) | 0.75 (0.60–0.95) |
| Obesity (BMI ≥ 25) | 0.71 (0.54–0.93) | 0.75 (0.57–0.99) |
| Ever-smokers | 0.73 (0.57–0.93) | 0.75 (0.57–0.99) |
| Positive HLA-B27 (vs. negative) | 1.63 (1.03–2.56) | 1.64 (1.03–2.61) |
| AS (vs. nr-axSpA) | 0.66 (0.45–0.96) | 0.71 (0.48–1.05) |
| TNFi naïve | 0.98 (0.73–1.31) | 0.99 (0.72–1.37) |
| Baseline ASDAS-CRP, unit | 0.77 (0.69–0.87) | 0.86 (0.75–0.99) |
| Longitudinal variable |  |  |
| Follow-up time (vs. interval in the one-year follow-up) | Reference | Reference |
| Interval in the two-year follow-up | 0.98 (0.82–1.17) | 0.60 (0.45–0.81) |
| Interval in the three-year follow-up | 1.06 (0.83–1.36) | 0.63 (0.44–0.90) |
| ASDAS-CRP_t-1_, unit | 0.92 (0.86–0.98) | 0.81 (0.69–0.94) |
| Concomitant NSAID use during the interval | 0.44 (0.35–0.56 | 0.42 (0.33–0.55) |
| Concomitant sulfasalazine use during the interval | 0.31 (0.11–0.94) | 0.28 (0.08–0.90) |
| Concomitant MTX use during the interval | 1.37 (0.76–2.44) | 1.62 (0.81–322) |
| Group according to the interval DQ |  |  |
| Control group (DQ = 100) | Reference | Reference |
| Mild-tapering group (50 ≤ DQ < 100) | 1.21 (0.98–1.48) | 0.90 (0.57–1.44) |
| Heavy-tapering group (DQ < 50) | 0.44 (0.22–0.88) | 0.26 (0.08–0.86) |
| QIC of the model |  | 1970.124 |

AS, ankylosing spondylitis; ASDAS, Ankylosing Spondylitis Disease Activity Score; BMI, body mass index; CI, confidence interval; CRP, C-reactive protein; DQ, dose quotient; HLA, human leukocyte antigen; LDA, low disease activity; MTX, methotrexate; nr-axSpA, non-radiographic axial spondyloarthritis; NSAID, non-steroidal anti-inflammatory drug; OR, odds ratio; QIC, quasi-likelihood under the Independence model Criterion; TNFi, tumor-necrosis factor inhibitor.

^*^, The model included all variables irrespective of the statistical significance in the univariable model.

**Supplementary table S4.** Effect of tapered DQ on the achievement of consecutive ASDAS-ID in the subgroup of patients who completed 3-year follow-up

|  | Univariable model  OR (95% CI)  (n = 757–776) | Baseline model^*^  OR (95% CI)  (n = 748) | Longitudinal model^⁋^  OR (95% CI)  (n = 748) |
| --- | --- | --- | --- |
| Baseline variable |  |  |  |
| Age, 10 years | 0.96 (0.81–1.13) | ^‡^ | ^‡^ |
| Female sex | 1.47 (0.90–2.41)/ | 1.11 (0.60–2.05) | 1.00 (0.53–2.00) |
| Disease duration, 10 years | 0.85 (0.61–1.19) | ^‡^ | ^‡^ |
| Obesity (BMI≥25) | 0.80 (0.48–1.32) | ^‡^ | ^‡^ |
| Ever-smokers | 0.59 (0.39–0.90)/ | 0.63 (0.39–1.02) | 0.63 (0.39–1.01) |
| Positive HLA-B27 (vs. negative) | 1.18 (0.59–2.36) | ^‡^ | ^‡^ |
| Definite radiographic sacroiliitis | 0.69 (0.37–1.29) | ^‡^ | ^‡^ |
| TNFi naïve | 0.81 (0.50–1.31) | ^‡^ | ^‡^ |
| Baseline ASDAS-CRP, unit | 0.66 (0.54–0.81)/ | 0.67 (0.55–0.83) | 0.72 (0.58–0.90) |
| Longitudinal variable |  |  |  |
| Follow-up time (vs. interval in the 1-year follow-up) | Reference | ^†^ | ^‡^ |
| Interval in the 2-year follow-up | 0.93 (0.70–1.23) | ^†^ | ^‡^ |
| Interval in the 3-year follow-up | 1.10 (0.83–1.45) | ^†^ | ^‡^ |
| ASDAS-CRP _t-1_, unit | 0.87 (0.78–0.98) | ^†^ | 0.93 (0.79–1.08) |
| Concomitant NSAID use during the interval | 0.42 (0.29–0.61) | ^†^ | 0.47 (0.31–0.70) |
| Concomitant MTX use during the interval | 1.05 (0.40–2.77) | ^†^ | ^‡^ |
| Group according to the interval DQ |  |  |  |
| Control group (DQ=100) | Reference | Reference | Reference |
| Mild-tapering group (50≤DQ<100) | 1.01 (0.76–1.35) | 0.95 (0.70–1.28) | 0.86 (0.42–1.78) |
| Heavy-tapering group (DQ<50) | 0.83 (0.33–2.06) | 0.80 (0.32–2.01) | 0.35 (0.08–1.47) |
| QIC of the model | 924.564 | 888.392 | 857.960 |

BASDAI, Bath Ankylosing Spondylitis Activity Index; BMI, body mass index; CI, confidence interval; CRP, C-reactive protein; DQ, dose quotient; HLA, human leukocyte antigen; MTX, methotrexate; NSAID, Non-steroidal anti-inflammatory drug; OR, odds ratio; QIC, Quasi-likelihood under the Independence model Criterion; TNFi, tumour-necrosis factor inhibitor.

^*^, The model was adjusted for baseline clinical factors showing a relevant association (P < 0.2) with the outcome in the univariable model.

^†^, Does not included in the model.

^‡^, Does not included in the model because its association with the outcome was not relevant (P ≥ 0.2)

^⁋^, The model was adjusted for covariates in the baseline model and longitudinal factors with a relevant association (P < 0.2) with the outcome in the univariable model.
